# Blood Brain-Derived Neurotrophic Factor (BDNF) and Major Depression: Do We Have a Translational Perspective?

**DOI:** 10.3389/fnbeh.2021.626906

**Published:** 2021-02-12

**Authors:** Beatrice Arosio, Franca Rosa Guerini, Richard C. Oude Voshaar, Ivan Aprahamian

**Affiliations:** ^1^Geriatric Unit, Fondazione Ca' Granda, IRCCS Ospedale Maggiore Policlinico, Milan, Italy; ^2^Department of Clinical Sciences and Community Health, University of Milan, Milan, Italy; ^3^IRCCS Fondazione Don Carlo Gnocchi, ONLUS, Milan, Italy; ^4^Department of Psychiatry, University of Groningen, University Medical Center Groningen, Groningen, Netherlands; ^5^Group of Investigation on Multimorbidity and Mental Health in Aging (GIMMA), Geriatrics Division, Internal Medicine Department, Faculty of Medicine of Jundiaí, Jundiaí, Brazil

**Keywords:** brain-derived neurotrophic factor, major depression, neuroplasticity, neurotrophin, review

## Abstract

Major depressive disorder (MDD) affects millions of people worldwide and is a leading cause of disability. Several theories have been proposed to explain its pathological mechanisms, and the “neurotrophin hypothesis of depression” involves one of the most relevant pathways. Brain-derived neurotrophic factor (BDNF) is an important neurotrophin, and it has been extensively investigated in both experimental models and clinical studies of MDD. Robust empirical findings have indicated an association between increased BDNF gene expression and peripheral concentration with improved neuronal plasticity and neurogenesis. Additionally, several studies have indicated the blunt expression of BDNF in carriers of the Val66Met gene polymorphism and lower blood BDNF (serum or plasma) levels in depressed individuals. Clinical trials have yielded mixed results with different treatment options, peripheral blood BDNF measurement techniques, and time of observation. Previous meta-analyses of MDD treatment have indicated that antidepressants and electroconvulsive therapy showed higher levels of blood BDNF after treatment but not with physical exercise, psychotherapy, or direct current stimulation. Moreover, the rapid-acting antidepressant ketamine has presented an early increase in blood BDNF concentration. Although evidence has pointed to increased levels of BDNF after antidepressant therapy, several factors, such as heterogeneous results, low sample size, publication bias, and different BDNF measurements (serum or plasma), pose a challenge in the interpretation of the relation between peripheral blood BDNF and MDD. These potential gaps in the literature have not been properly addressed in previous narrative reviews. In this review, current evidence regarding BDNF function, genetics and epigenetics, expression, and results from clinical trials is summarized, putting the literature into a translational perspective on MDD. In general, blood BDNF cannot be recommended for use as a biomarker in clinical practice. Moreover, future studies should expand the evidence with larger samples, use the serum or serum: whole blood concentration of BDNF as a more accurate measure of peripheral BDNF, and compare its change upon different treatment modalities of MDD.

## Introduction

Depression affects 264 million people globally according to World Health Organization estimates and is a leading cause of disability (GBD 2017, Disease and Injury Incidence and Prevalence Collaborators, 2018). Major depressive disorder (MDD), the most common presentation of this illness, has a prevalence in 12 months and throughout life of 10.4 and 20.6%, respectively (Hasin et al., 2018). Furthermore, antidepressants are the most commonly used drugs, as ~13% of Americans 12 years or older are prescribed these antidepressants (U. S. Department of Health Human Services, 2017). Most episodes of depression throughout life are classified as moderate or severe and result in great loss of quality of life and productive years (Hasin et al., 2018). Of the total episodes, 12% are hospitalized at some point (Hasin et al., 2018). Nonetheless, the pathophysiology of depression is multifactorial and not totally understood. Several pathophysiological systems are implicated in MDD, such as the immune system, autonomic nervous system, and hypothalamic–pituitary–adrenal axis, as well as primary brain systems, including the monoaminergic brain circuitries and the neurotrophic support pathway (Krishnan and Nestler, 2008). However, no biomarkers have been clearly identified as being capable of improving diagnostic accuracy, guiding treatment selection or response, or predicting prognosis consistently.

In the last 70 years, the “monoamine hypothesis” has been a dominant biochemical theory for depression. However, lack of response to antidepressants restoring monoamines in several patients and the clinical latency of response of several weeks consisted of some of the arguments questioning this theory (Hindmarch, 2002). Almost two decades ago, the “neurotrophin hypothesis of depression” was proposed by Duman et al. (1997) as a putative biological mechanism for depression. This hypothesis postulated that depression was due to dysfunctional neurogenesis in brain regions responsible for emotion and cognition (Duman and Monteggia, 2006). According to this hypothesis, the expression of neuronal growth factors (neurotrophins) is decreased when facing a stressor. Neurotrophins are proteins that induce the survival, development, and differentiation of neurons. This stress-related decrease in neurotrophic support reduces hippocampal neurogenesis and results in neuronal atrophy and the loss of glial cells, whereas antidepressant treatment could restore neurotrophic support by upregulating neuronal growth factors. Among the many known neurotrophins, including nerve growth factor, neurotrophin-3, and neurotrophin-4, brain-derived neurotrophic factor (BDNF) is the most abundant and widely neurotrophic growth factor in the central nervous system. It regulates nervous system development and survival during the entire life span through the activation of three signaling pathways, MAPK, phospholipase C-γ, and phosphoinositide 3-kinase, which bind to its receptor tropomyosin receptor kinase B (TrkB) (Saarelainen et al., 2003). In particular, BDNF modulates cognitive functions through its capacity to modulate neurite outgrowth, neuronal differentiation and survival, growth, and guidance of axons and dendrites, synaptic plasticity, long-term potentiation, and neurotransmitter release (Huang and Reichardt, 2001; Patapoutian and Reichardt, 2001; Poo, 2001; Chao, 2003; Tapia-Arancibia et al., 2008).

The neurotrophic hypothesis of depression is heavily based on the correlation between lower levels of BDNF and a higher frequency of depression, depressive symptomatology, neuronal loss, and cortical atrophy, and the restoration of the BDNF effect is linked to antidepressants (Martinowich et al., 2007). This hypothesis has stimulated many studies, which unfortunately revealed contradictory findings from experimental studies to clinical trials and questioned further translational evidence for the clinical application of quantification of BDNF in serum, whole blood, or plasma for the diagnosis, monitoring, and prognosis of MDD. Important questions remain fragmented across experimental and genotype studies, clinical trials, and meta-analyses, and the correct interpretation of these findings may avoid noise and misunderstanding surrounding peripheral BDNF concentrations in MDD. Most previous integrative reviews on BDNF in MDD date more than 10 years ago (Groves, 2007; Martinowich et al., 2007). At that time, BDNF was a potential target for antidepressant therapy, and the BDNF molecular pathway (from pro-BDNF to mature protein) was under debate. Since then, at least five important meta-analyses regarding the serum BDNF response to antidepressants (Brunoni et al., 2008; Sen et al., 2008; Bocchio-Chiavetto et al., 2010; Molendijk et al., 2014; Zhou et al., 2017) have also been published aiming to identify peripheral biomarkers for antidepressant therapy. More recent reviews have mainly focused on BDNF-related antidepressant actions, specifically on rapid-acting antidepressants or only experimental evidence, but do not approach several other treatment modalities in MDD or simultaneously integrate basic science and clinical trials (Duman et al., 2019; Yang et al., 2020). This review aims first to summarize current evidence (see Box 1 for the search strategy assumed) of BDNF function, genetics, epigenetics, and expression. Second, we review clinical and therapeutic evidence, beyond antidepressants, regarding BDNF concentration in MDD and the potential role of BDNF as a biomarker in clinical practice.

Box 1 **Search strategy for this review.**

For this review, we have searched the PubMed primarily for narrative reviews, systematic reviews, meta-analyses, and clinical trials published in English between January 1, 1995, and September 1, 2020, using the search terms “depression” and “depressive” cross-referenced with the terms “neurotrophins”, “brain-derived neurotrophic factor” and “BDNF”. To improve the strength of the evidence reviewed here, we assembled two professionals with experience in experimental and genetic research and two professionals with both clinical and research activities in mood disorders, mainly focused on depressive disorders, to write this review. Also, we primarily focused on systematic reviews and meta-analyses to reduce publication and selection bias of the published evidence in this non-systematic review. Reference lists from these publications were searched and added in our review if judged relevant. All authors followed this strategy, and any disagreement of selection criteria was solved through consensus opinion among the authors.

## BDNF Function

BDNF is part of a family of structurally related peptides, named neurotrophins, which are able to interact with two classes of receptors expressed on cell membrane surfaces, namely, tropomyosin receptor kinase (Trk) A–C, which binds to diverse neurotrophins, and the p75 neurotrophin receptor (p75NTR) (Chao, 2003). In general, both autocrine and paracrine mechanisms are used for the synthesis and action of neurotrophins by different cell types in the nervous, immune, and endocrine systems (Vega et al., 2003). The most important neurogenesis and neuroprotectant roles come from the BDNF activity over TrkB receptors (Ibáñez, 1995; Fossati et al., 2004).

First, BDNF is synthesized as a pro-protein (pro-BDNF) in the brain, especially in the hippocampus and hypothalamus (Chao, 2003). Next, pro-BDNF is cleaved to form mature BDNF and pro-peptide (the pro-BDNF N-terminal fragment) with specific biological functions. BDNF is produced by mature neurons and glial cells and released together with other neurotrophins after neuron depolarization (Aloe et al., 2002; Lessmann et al., 2003; Castrén, 2004). It is believed that an imbalance of pro-BDNF and mature BDNF can cause neuronal degeneration and behavioral impairment (Cuello et al., 2010; Rosso et al., 2017).

## Genetics of BDNF

**Figure 1:**
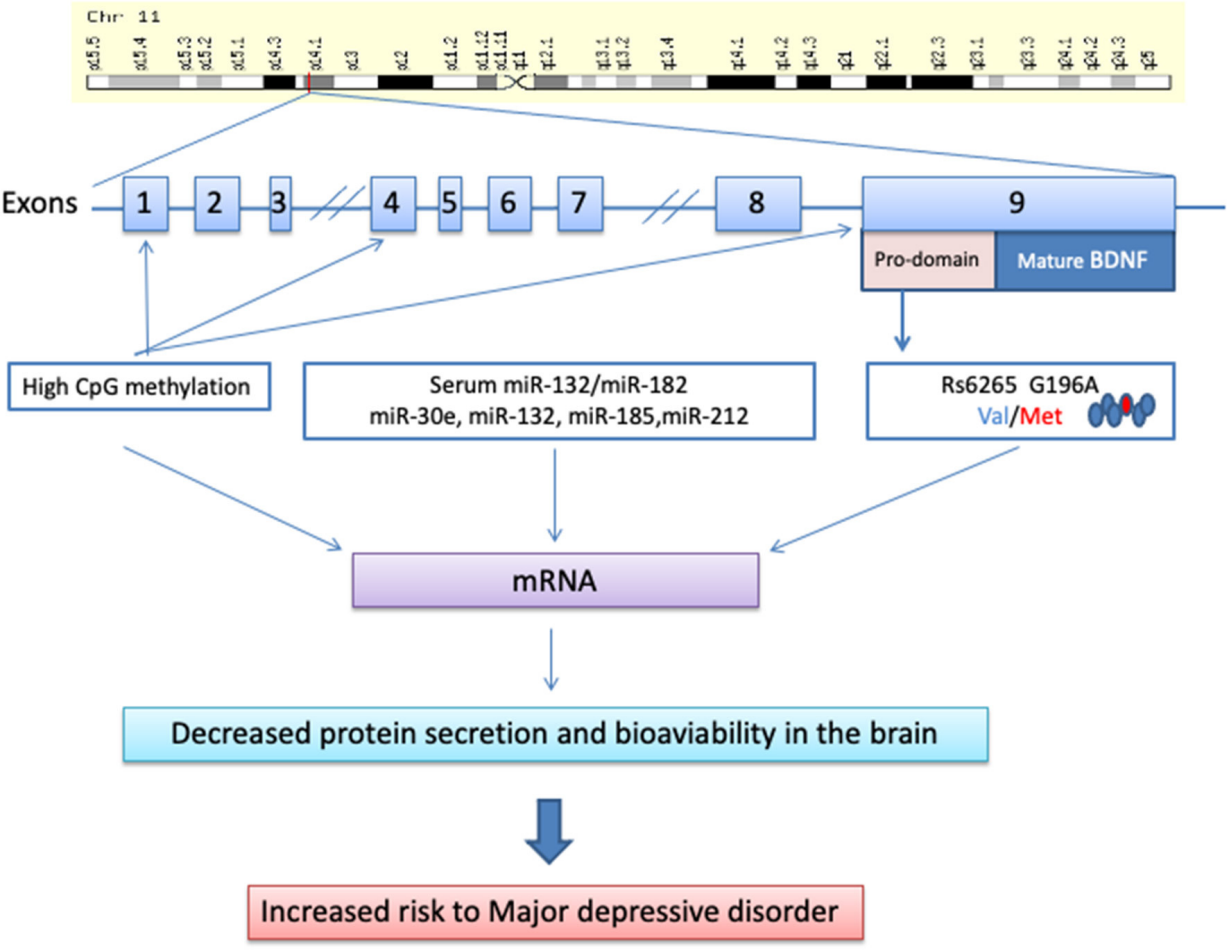
Molecular biology of the brain-derived neurotrophic factor and its way toward major depressive disorder.

The human *BDNF* gene is located on chromosome 11p13 and consists of 11 exons and 9 functional promoters that are tissue-specific and brain region–specific (Pruunsild et al., 2007). This gene has many variants, and several single-nucleotide polymorphisms (SNPs) have been described so far (Licinio et al., 2009). The most studied *BDNF* SNP is a transition from G to A at position 196 in the exon IX gene (rs6265 or G196A polymorphism), resulting in an amino acid substitution at codon 66 (valine [Val] to methionine [Met]) in the precursor BDNF peptide, also known as Val66Met polymorphism (Pruunsild et al., 2007; Tsai, 2018). The functional role of this SNP is to modulate the secretion of BDNF by neurons (Egan et al., 2003). Conflicting results arise regarding the possible effects of this *BDNF* gene polymorphism in depressive disorders. Common points of view assume that the *BDNF* Val66Met polymorphism is not associated with depressive disorders *per se* (Verhagen et al., 2010; Gyekis et al., 2013), but age, sex, environment, ethnicity, and gene–gene interaction may influence its role in depression (Figure 1).

### Val66Met and Sex Environment and Ethnicity

Interestingly, in a large meta-analysis, the Met allele significantly increased the risk of depression in men but not in women (Verhagen et al., 2010). A possible explanation derives from bioimaging studies showing that the hippocampus is larger in women than in men when adjusted for total brain size (Goldstein et al., 2001) and that the hippocampi of women differ significantly in neurochemical make-up (Madeira and Lieberman, 1995), as well as reactivity to stressful situations (Cahill, 2006). As demonstrated in animal models, chronic stress causes damage to the hippocampus in males but does so far less in females (McEwen, 2000).

As stress leads to decreased levels of BDNF expression in the hippocampus, and the *BDNF* gene plays a role in the survival of neuronal cells, connectivity, and plasticity, a more efficiently expressed *BDNF* allele might protect the brain against hippocampal damage after stress or at least might render an individual less vulnerable to the effects of sustained stress (Smith et al., 1995).

Given these findings, the *BDNF* Val66Met polymorphism may play a larger role in the neurobiology of depression in men than in women (Verhagen et al., 2010). Moreover, the different capacity to cope with stress in men compared with women may be due to different interplay between genetic and environmental factors in the two sexes (Becker et al., 2007). For example, the association between the *BDNF* gene profile and childhood stressful life events is present only in men (van Oostrom et al., 2012), as is the effect of BDNF polymorphisms on the relationship between physical activity and cognitive performance (Watts et al., 2018).

Under these premises, we must consider that the interaction between the *BDNF* Val66Met polymorphism and MDD also depends on the geographic origin. Indeed, a meta-analysis showed a stronger association between this polymorphism and MDD in a white population than in an Asian population (Zhao et al., 2018). These findings suggest that the interaction between the *BDNF* Val66Met polymorphism and life stress in depression may differ across cultural contexts and that *BDNF* gene variants may exert a different role in different ancestries (Yang et al., 2020).

Moreover, the methodologies used to measure stress are important factors able to influence the results. For example, in children, the so-called early life stress is measured differently in the various studies (e.g., the time of occurrence) (Kim et al., 2007; Chen et al., 2012). Finally, the association between depression and *BDNF* gene profiles is much more evident in those studies using in-person interviews or objective measures to assess stress (e.g., trained investigators) than in studies using self-report methods (Verhagen et al., 2010).

### Val66Met and Gene by Gene Interaction

Gene-by-gene interactions have been hypothesized to contribute to the etiology of depression (Kaufman et al., 2006). The most significant *BDNF* gene interaction was reported with the serotonin transporter-linked polymorphic region (*5-HTTLPR*) in the promoter of the *SLC6A4* gene that encodes 5-HTT (Harkness et al., 2015; Bleys et al., 2018). *5-HTTLPR* is a 43-base-pair insertion/deletion polymorphism that creates two allelic forms, the long (L) allele and the short (S) allele associated with decreased 5-HTT expression and serotonin reuptake (Lesch et al., 1996). A cross-sectional study demonstrated that the Met allele of the *BDNF* gene might interact with two short alleles in the promoter region of the *5-HTTLPR* increasing the risk for depression in maltreated children (Kaufman et al., 2006). Indeed, a study has suggested that the serotonin (*5-HT*)–*BDNF* systems act synergistically on synaptic plasticity and neurogenesis in brain areas implicated in depression (Martinowich and Lu, 2008).

The *BDNF* gene is also required for *dopamine D*_3_ *receptor* (*DRD3*) expression (Guillin et al., 2001). The most frequently studied polymorphism is *DRD3* Ser9Gly (rs6280), which causes a serine (Ser)-to-glycine (Gly) substitution increasing the affinity for binding to dopamine (Lundstrom and Turpin, 1996; Bombin et al., 2008). Interestingly, *DRD3* Ser9Gly has been described as interacting with *BDNF* Val66Met in the development of anxiety disorder comorbidity in patients with bipolar disorder II (Chang et al., 2013).

## BDNF Epigenetics

DNA methylation of the *BDNF* gene has been widely investigated, showing increased *BDNF* methylation levels in patients with MDD (Schröter et al., 2020). In particular, high methylation of CpG sites in exon I and low serum levels of BDNF were associated with MDD (D'Addario et al., 2013; Carlberg et al., 2014) and depression in patients characterized by bipolar II disorders (Dell'Osso et al., 2014). A significantly higher methylation level was also observed at the 217 CpG site in *BDNF* exon IX in MDD patients than in healthy controls (Hsieh et al., 2019). In contrast, lower methylation levels at the 327 CpG site and 362 CpG site were observed in the same patients (Hsieh et al., 2019). Interestingly, late-life depression was also associated with elevated *BDNF* methylation of specific CpG sites within *BDNF* promoters I and IV (Januar et al., 2015).

Another important epigenetic effect is played by microRNAs, endogenous small non-coding RNAs, which post-transcriptionally regulate the expression of several genes targeting mRNAs for cleavage and/or translational repression. Interestingly, in depressed patients, there is an inverse relationship between serum BDNF levels and miR-132/miR-182 levels (Liu et al., 2016). Moreover, the serum levels of miR-30e, miR-132, miR-185, and miR-212 were significantly increased in MDD patients compared with healthy controls (Lin et al., 2017).

## BDNF Expression and Blood BDNF in Neuropsychiatric Diseases

Gene expression of *BDNF* has been found in the brain and other non-nervous compartments including human lymphocytes and monocytes (Kerschensteiner et al., 1999). Peripherally, BDNF can be identified in blood and saliva samples (Lipps, 2000; Mandel et al., 2009; Tirassa et al., 2012). In humans, determinants of serum BDNF levels include sampling factors (e.g., fasting vs. non-fasting, time of sampling, duration of sample storage), sociodemographics (sex, age, urban, vs. rural), lifestyle (use of smoking or alcohol), and even seasonality (Bus et al., 2011, 2012; Molendijk et al., 2012). Unfortunately, most clinical studies have not been fully adjusted for these potentially confounding characteristics. In addition, platelets can store BDNF and act as a “buffer system” regulating the peripheral concentration of BDNF (Serra-Millàs, 2016). During aging, the central and peripheral levels of BDNF are reduced (Lommatzsch et al., 2005), particularly in older persons affected by mood disorders and cognitive impairment (Pal et al., 2014). Nonetheless, the manifestations and severity of several neurodegenerative and psychiatric disorders are correlated with BDNF levels (Bersani et al., 2000; Gelfo et al., 2011). However, BDNF levels can be susceptible to stimuli-related alterations in healthy conditions (Tirassa et al., 2012).

Animal studies proposed that BDNF could cross the blood–brain barrier in both directions, as after BDNF was injected in the brain, it was removed to the periphery (Pan et al., 1998); however, this evidence is highly questionable based on several studies showing a high concentration of brain BDNF in mice, but without peripheral BDNF detection (Radka et al., 1996; Chacón-Fernández et al., 2016). In humans, peripherally detectable BDNF is mostly related to its presence in megakaryocytes and platelets, but these cells do not have nuclei to synthetize BDNF (Chacón-Fernández et al., 2016). However, BDNF is mainly stored in platelets, which explains the ~200-fold difference between serum and plasma BDNF, probably due to BDNF release during the coagulation process (Karege et al., 2002, 2005). Whether peripheral BDNF is only stored in platelets from brain production or is derived from peripheral production remains to be better addressed. During aging, the central and peripheral levels of BDNF are reduced (Lommatzsch et al., 2005), particularly in older persons affected by mood disorders and cognitive impairment (Pal et al., 2014). Reduced levels of BDNF have been described as being involved in the pathogenesis of Alzheimer disease (Huang and Reichardt, 2001). Indeed, the gene and protein expression of BDNF is severely reduced in the hippocampus and temporal and frontal cortex regions in Alzheimer disease brain (Phillips et al., 1991; Hock et al., 2000; Patapoutian and Reichardt, 2001; Fahnestock et al., 2002; Michalski and Fahnestock, 2003). Interestingly, in these patients, the postmortem cortex levels of BDNF were correlated with circulating serum BDNF levels, and the protein levels were associated with cognitive decline evaluated by the Mini Mental State Examination score (Poo, 2001; Michalski and Fahnestock, 2003; L. Tapia-Arancibia et al., 2008).

Regarding MDD, there are several studies with inconsistent results (Monteleone et al., 2008; Fernandes et al., 2009; Kreinin et al., 2015), possibly because the majority of them have not investigated the BDNF system across molecular levels. Moreover, longitudinal studies to determine whether altered BDNF levels are state or trait markers of disease are sparse (Karege et al., 2005; Monteleone et al., 2008; Piccinni et al., 2008; Kreinin et al., 2015).

MDD patients presented lower mRNA (from blood mononuclear cells) and serum concentrations of BDNF than healthy controls (Karege et al., 2005; Lee and Kim, 2010; Hsieh et al., 2019; Schröter et al., 2020). In particular, the serum BDNF levels appear to be very low in patients who have attempted suicide (Lee and Kim, 2010; Ai et al., 2019). On the other hand, other studies did not show different serum levels of BDNF in MDD patients compared with controls (Duman et al., 1997), and others also described an improvement in BDNF levels in women but not in men after antidepressant treatment (Huang and Hung, 2009; Polyakova et al., 2015b; Martinotti et al., 2016; Lin et al., 2017). Several mechanisms could be hypothesized regarding the impairment of DNA expression and decreased serum BDNF production. As reported previously, the different promoter methylation statuses in exon IX (Hsieh et al., 2019) and in exon I may be one of the mechanisms involved (D'Addario et al., 2013; Schröter et al., 2020). The biological effects of BDNF are mediated by the transmembrane tropomyosin-related kinase B (TrkB) receptor, and a downregulation of the TrkB signaling pathway has been discovered in MDD (Tsai, 2004). Finally, it has been suggested that the decreased serum and plasma BDNF levels (but not in whole-blood BDNF) in MDD patients are related to mechanisms of BDNF release and secretion independent of platelet reactivity (Karege et al., 2005).

## Clinical and Treatment Perspectives

The neurotrophin hypothesis of depression proposes that most of the antidepressant effect comes from the increase in BDNF expression and concentration, which improves neuronal plasticity (Park and Poo, 2013). This latter process is particularly important in sites of cell proliferation, such as the subventricular and subgranular zones of the dentate gyrus, which is involved in maintaining stable mood (Gross, 2000). Experimental findings in rats showed that a reduction in BDNF impaired neurogenesis and induced depressive-like behavior, antidepressants increased brain BDNF, and finally, intrahippocampal injections of the neurotrophin diminished depressive-like symptoms (Shirayama et al., 2002; Deltheil et al., 2009; Taliaz et al., 2010; Kavalali and Monteggia, 2012). Translational evidence came from postmortem studies showing low BDNF level and its receptor, TrkB, in region-specific depressed brains (Kozicz et al., 2008; Bernard et al., 2011; Ray et al., 2011). Taken together, this evidence stimulated seminal clinical studies showing low serum levels of BDNF in depressed individuals with a direct proportional relation between BDNF concentration and depressive symptomatology (Karege et al., 2002), as well as an increase in serum BDNF levels during antidepressant treatment (Shimizu et al., 2003).

Clinical studies showed heterogeneous results. Inconsistencies were seen between BDNF levels and depressive symptom severity. Generally, patients with suicide attempts and a high risk of suicide showed lower levels of serum or plasma BDNF (Dawood et al., 2007; Deveci et al., 2007; Kim et al., 2007). Antidepressant treatment restored serum BDNF levels in several studies (Brunoni et al., 2008), but a variety of times of response and types of drug were observed. Two previous studies showed that selective serotonin receptor inhibitor (SSRI) and serotonin and norepinephrine receptor inhibitor (SNRI) treatment for 2 months increased serum BDNF (Gonul et al., 2005; Yoshimura et al., 2007). Notwithstanding, a study with sertraline and escitalopram observed an increase in serum BDNF levels only after 6 months (Matrisciano et al., 2009). In this study, no change in BDNF levels was observed using venlafaxine. Another important issue is the medium in which BDNF is evaluated, i.e., serum or plasma. Usually, clinical studies have indicated an increase in plasma and serum BDNF levels in MDD antidepressant-treated patients after 45–60 days (Yoshimura et al., 2007; Lee and Kim, 2008). However, a small sample size study with a follow-up of 12 months found a variation in serum and plasma BDNF response to antidepressants, with the former persistently low and no changes regarding the latter (Piccinni et al., 2008).

To date, more than 50 studies have been performed aiming to investigate whether BDNF could constitute a depressive episode biomarker and to what magnitude it could signal an antidepressant response. Most of these studies' findings are represented in a large meta-analysis aimed at systematically reviewing the effect size of antidepressants on BDNF serum levels in 9,484 (in 55 studies) healthy controls, antidepressant-free MDD patients, and antidepressant-treated MDD patients (Molendijk et al., 2014). The random-effects sizes showed that antidepressant-free depressed patients had lower BDNF concentrations than those of healthy controls (effect size of −0.71) and those of antidepressant-treated depressed patients (effect size of −0.56). The BDNF levels were not different between antidepressant-treated patients and healthy controls (effect size of 0.07, *p* = 0.52). The BDNF concentrations and depressive symptom severity were negatively correlated in antidepressant-free depressed patients (*r* = −0.19; *p* < 0.001) but not in antidepressant-treated depressed patients or healthy controls.

This systematic review confirmed previous results from three other meta-analyses (*n* = 968; 11 studies with low to medium sample sizes), which found an antidepressant restoration (effect size of −1) of low serum BDNF in treatment-free depressed individuals (effect size of −1) (Brunoni et al., 2008; Sen et al., 2008; Bocchio-Chiavetto et al., 2010). However, the meta-analysis performed by Molendijk et al. (2014) revealed a large amount of heterogeneity (e.g., in severity of depression) and publication bias (e.g., larger samples and more recent publications with smaller differences between groups). Nonetheless, correcting effect size estimates for publication bias resulted in attenuated values for low levels of BDNF and its resultant antidepressant effect, being almost half of the previous values observed in the study. However, the association between BDNF concentration and depressive symptom severity disappeared. Moreover, most of the studies included were underpowered (median sample size of 36 patients).

A more recent systematic review and network meta-analysis found a significant effect of antidepressants on increasing BDNF levels [standardized mean difference (SMD) = 0.62; 95% confidence interval (CI) = 0.31–0.94, *Z* = 3.92, *p* < 0.0001] (Zhou et al., 2017). An increase in BDNF levels over time was also associated with a significant decrease in the Hamilton Depression Rating Scale score (SMD = 2.78, 95% CI = 2.31–3.26, *Z* = 11.57, *p* < 0.00001). SNRIs showed a higher effect size than SSRIs (0.92 vs. 0.68). Moreover, four antidepressants presented more than one study and were analyzed individually regarding their role in increasing BDNF levels. Only sertraline showed significantly increased BDNF levels after treatment (SMD = 0.53, 95% CI = 0.13–0.93, *Z* = 2.62, *p* = 0.009), whereas venlafaxine, paroxetine, and escitalopram did not. In general, antidepressants had a significant effect on the increase in BDNF concentration after 8 weeks. Only serum levels of BDNF were correlated with antidepressant treatment, whereas plasma BDNF was not. A high degree of heterogeneity was also found in this systematic review ranging from 83 to 85%. Although these meta-analyses carry some methodological limitations inherent to this type of study, collectively, yielded evidence raises concern about the utility of serum BDNF as a clinical biomarker for MDD or a predictor for antidepressant efficacy, and further investigations are necessary to recommend the usage of BDNF levels to monitor antidepressant treatment.

Central and peripheral levels of BDNF are highly correlated (Klein et al., 2011). As stated before, the most peripheral BDNF is stored in platelets (almost 100%), resulting in very low levels of free BDNF in the plasma (Fujimura et al., 2002). As platelets do not cross the blood–brain barrier, plasma levels are more associated with brain levels of BDNF (Radka et al., 1996). Additionally, during the process of preparing blood samples to measure serum BDNF, coagulation and centrifugation processes result in platelet release of BDNF, increasing its level (Fujimura et al., 2002). Several technical issues influence serum or plasma BDNF levels such as clotting time, bioassays, temperature, and a second centrifugation to correct plasma levels, among others (Maffioletti et al., 2014; Amadio et al., 2017; Gejl et al., 2019). Most importantly, platelet alterations are observed in MDD, and several drugs such as antidepressants and antiaggregating medications (e.g., clopidogrel) influence BDNF platelet release (Serra-Millàs, 2016). Although plasma and serum BDNF are used as equivalents in clinical MDD research, they should not be viewed as such. In this sense, we believe that serum BDNF is more reliable for clinical practice and clinical studies, especially when evaluating the serum: whole blood concentration as previously recommended (Karege et al., 2002, 2005).

Rapid-acting antidepressants are mainly represented by low-dose intravenous infusion of ketamine, an *N*-methyl-d-aspartate receptor (NMDA-R) antagonist, which produces a fast and significant reduction in suicidal ideation and depressive symptoms in MDD patients (Wilkinson et al., 2018). An increase in synaptic BDNF levels in γ-aminobutyric acid interneurons appears to be a major mechanism of action of ketamine (Zanos and Gould, 2018). Interestingly, the BDNF levels increased after an average time of 4 h after a low-dose infusion of ketamine, completely different from the evidence with other antidepressants, which require a more prolonged time (Autry et al., 2011; Kavalali and Monteggia, 2012; Haile et al., 2014). Additionally, the presence of the *BDNF* Val66Met polymorphism may blunt the antidepressant and antisuicidal effects of ketamine (Laje et al., 2012). The rapid and direct effect of BDNF linked to ketamine is reinforced by the lack of the same benefit for other NMDA-R antagonists (memantine), a blunt in antidepressant action of ketamine in deletion mutant mice, and a blocked action after infusion of anti-BDNF in the medial prefrontal cortex (Autry et al., 2011; Gideons et al., 2014; Lepack et al., 2014). Robust clinical evidence is needed to confirm these findings.

Notwithstanding, other treatment alternatives to MDD were also investigated regarding a potential increase in peripheral BDNF concentration. This is the case for electroconvulsive therapy (ECT), which is a potent catalyst for neurogenesis in the hippocampus of animal models (Pereira et al., 2007). ECT is an effective treatment for MDD, but its mechanism is still debatable, involving morphological brain modifications and modulation of monoaminergic neurotransmission, especially 5-HT_1A_ and 5-HT_2A_ receptors (Baldinger et al., 2014; Gryglewski et al., 2019). Previous experimental evidence showed that electroconvulsive shocks (ECT in animal models) resulted in higher levels of BDNF in rats (Polyakova et al., 2015a). Two recent meta-analyses included studies with MDD patients treated with ECT, in which pretreatment and posttreatment BDNFs were evaluated (Rocha et al., 2016; Luan et al., 2020). SMDs of 0.56 (95% CI = 0.17–0.96) and 0.695 (95% CI = 0.402–0.988) were observed between the studies pointing to an increase in serum BDNF after the ECT. However, long-term (>30 days) ECT was not evaluated (Luan et al., 2020), and a high heterogeneity was observed (Rocha et al., 2016). Most importantly, more than half of the studies with ECT used several combinations of antidepressants with ECT, which potentially alters BDNF concentration (Rocha et al., 2016). Moreover, a recent study showed that serum levels of BDNF increased after 1 day, 1 week, and 1 month of an ECT session, but BDNF concentrations were not associated with depressive symptomatology (Vanicek et al., 2019). Another ECT study found similar results, as the plasma BDNF levels and the Val66Met polymorphism neither predicted the effect of ECT nor were related to improvement in depression (Ryan et al., 2018). These findings hamper a potential mediating effect of BDNF by which ECT improves depression. Moreover, evidence from other non-invasive brain stimulation treatments was summarized in a 2015 meta-analysis (Brunoni et al., 2015). A total of 259 patients were included, mostly with treatment-resistant MDD. However, most patients received daily sessions of transcranial direct current stimulation on the left dorsolateral prefrontal cortex. No alteration in BDNF levels was found in any of the trials included, and a metaregression did not find predictive variables for this outcome. More importantly, no sign of heterogeneity or publication bias was found. A sensitivity analysis confirmed these findings.

Psychological treatment was also investigated regarding BNDF concentration and genetic profile. However, the results are less evident and less consistent. The serum levels of BDNF did not change after 16 weekly sessions of cognitive behavior therapy, and the plasma levels did not predict a response to 12 or 16 sessions of twice-weekly interpersonal therapy for depression in another study (Koch et al., 2009; da Silva et al., 2018). Moreover, a genome-wide meta-analysis did not associate the presence of the Val66Met polymorphism with psychological treatment outcomes for depression (Rayner et al., 2019). Finally, a recent trial evaluating the presence of the Val66Met polymorphism and pretreatment and posttreatment BDNF serum levels for both cognitive behavior therapy and interpersonal therapy in severe MDD patients did not find any significant association with outcomes after 6 months (Bruijniks et al., 2020). These results were not influenced by treatment modality or frequency (once or twice per week) of therapy. In this study, it was observed that high performance of working memory moderated the relation between higher levels of BDNF and lower posttreatment depression. This finding can shed some light on the mechanisms by which BDNF may influence psychotherapy results. Cognitive abilities are required in the psychotherapeutic process to reduce depressant stimulants and to cope with negative feelings (Bruijniks et al., 2020). Previously, cognitive domains such as memory performance (Azeredo et al., 2017) and attention (Mikoteit et al., 2015) were associated with BDNF levels, which are mainly expressed in the hippocampus, a region responsible for memory and inter-related cognitive functions in addition to the regulation of stress (Dranovsky and Hen, 2006).

Physical exercise is an evidence-based treatment strategy to improve depression with a moderate effect size (Schuch et al., 2016). Several mechanisms may explain the positive impact of physical exercise, including an increase in neurotrophic support (generally combined with an enriched environment). Animal studies have shown that physical exercise is associated with increased expression of BDNF in the hippocampus, which may improve memory performance and reduce depressive symptoms by promoting neurogenesis and neuronal differentiation (Hötting and Röder, 2013). Similar to antidepressants, there are patients with MDD who do not respond to physical activity practice at all (Dunn et al., 2005). One proposed theory is that the mechanism of action of exercise could involve the neurotrophin pathway, especially the BDNF. In humans, acute exercise increased serum BDNF, and chronic aerobic exercise produced higher concentrations of resting BDNF (Dinoff et al., 2016, 2017). A 2015 meta-analysis showed that BDNF was elevated after a single section of physical activity and after a regular program (Szuhany et al., 2015). Six studies were evaluated in a recent meta-analysis with conflicting results regarding the effect of physical exercise on resting concentrations of BDNF among MDD patients, and the peripheral BDNF concentrations were not increased after an exercise intervention (Dinoff et al., 2018). The small sample size in all studies, short-term follow-up, and a high level of heterogeneity were potential limitations in this systematic review. More recently, a secondary analysis of a pilot randomized controlled trial, which examined the combined effects of exercise and psychosocial treatment on depression symptoms, showed a non-significant mediating effect of BDNF (Szuhany and Otto, 2020). In this small trial, neither a moderate effect of exercise on changes in BDNF levels nor predicted changes in depressive symptomatology were sustained over time. Interestingly, the BDNF Val66Met polymorphism may moderate the impact of physical exercise on BDNF expression. In mouse depression models, exercises had no behavioral or neuroplastic effects in the presence of the BDNF Val66Met polymorphism (Ieraci et al., 2016). In an experiment with healthy people, the increase in serum BDNF levels after exercise was significantly less among carriers of the BDNF Val66Met polymorphism (Lemos et al., 2016). Whether the BDNF Val66Met polymorphism indeed moderates the effect of physical exercise on depressive symptoms in humans needs further exploration as studies thus far have produced inconsistent results (Mata et al., 2010; Gujral et al., 2014). Interestingly, a recent study showed that the positive impact of physical exercise on cognitive performance was significantly less among depressed patients with the BDNF Val66Met polymorphism than among those without this polymorphism (Pitts et al., 2020).

## Aging, Depression, and BDNF

Few studies have investigated the relationship between BDNF and late-life depression. Older adults with depression present more cognitive complaints, reduced hippocampal volume, and subcortical vascular abnormalities than younger individuals (Beekman, 2011). These characteristics could be associated with lower levels of BDNF or at least with the presence of the Val66Met polymorphism in geriatric depression. *BDNF* Val66Met polymorphism studies in MDD, especially in later life, are inconsistent. However, the presence of the *BDNF* Val66Met polymorphism was associated with geriatric depression in a previous meta-analysis of studies of late-life depression with low level of heterogeneity and publication bias (Pei et al., 2012). In late-onset depression, Met allele carriers were associated with higher white matter hyperintensity volumes but without obvious clinical relationships (Taylor et al., 2008). Interestingly, compared with Val carriers, healthy Met allele carriers showed worse episodic memory function, reduced hippocampal physiologic performance in functional magnetic resonance, and reduced prefrontal and hippocampal gray matter volume in previous studies (Egan et al., 2003; Pezawas et al., 2004; Szeszko et al., 2005; Bueller et al., 2006; Yu et al., 2009). Moreover, Met allele carriers also showed increased age-related impairment of hippocampal activation during encoding and retrieval tasks compared with Val carriers independent of structural or performance differences (Sambataro et al., 2010). Furthermore, in a large representative sample of older American veterans, the BDNF Val66Met polymorphism moderated the association between depression and lower cognitive performance (Pitts et al., 2020).

Most studies involving older adults with depression observed lower plasma or serum levels of BDNF (Diniz et al., 2010; Laske et al., 2010; Shi et al., 2010; Chu et al., 2012). However, in a Dutch cohort study of late-life MDD, no association was found between BDNF serum levels and cognitive performance (Dols et al., 2015). In this study, BDNF concentrations were not different between depressed older adults and controls. Most interestingly, one recent study found that the impact of BDNF serum levels on the course of late-life depression was conditional on the presence of both the use of SSRIs and a history of childhood trauma (Dimitriadis et al., 2019). Based on these results, the authors hypothesized that childhood trauma may permanently reduce (“blunt”) the responsiveness of the neurotrophic system to SSRI usage and that this responsiveness might be more important for depression course than the actual BDNF serum levels (Dimitriadis et al., 2019).

## Conclusions

**Figure 2:**
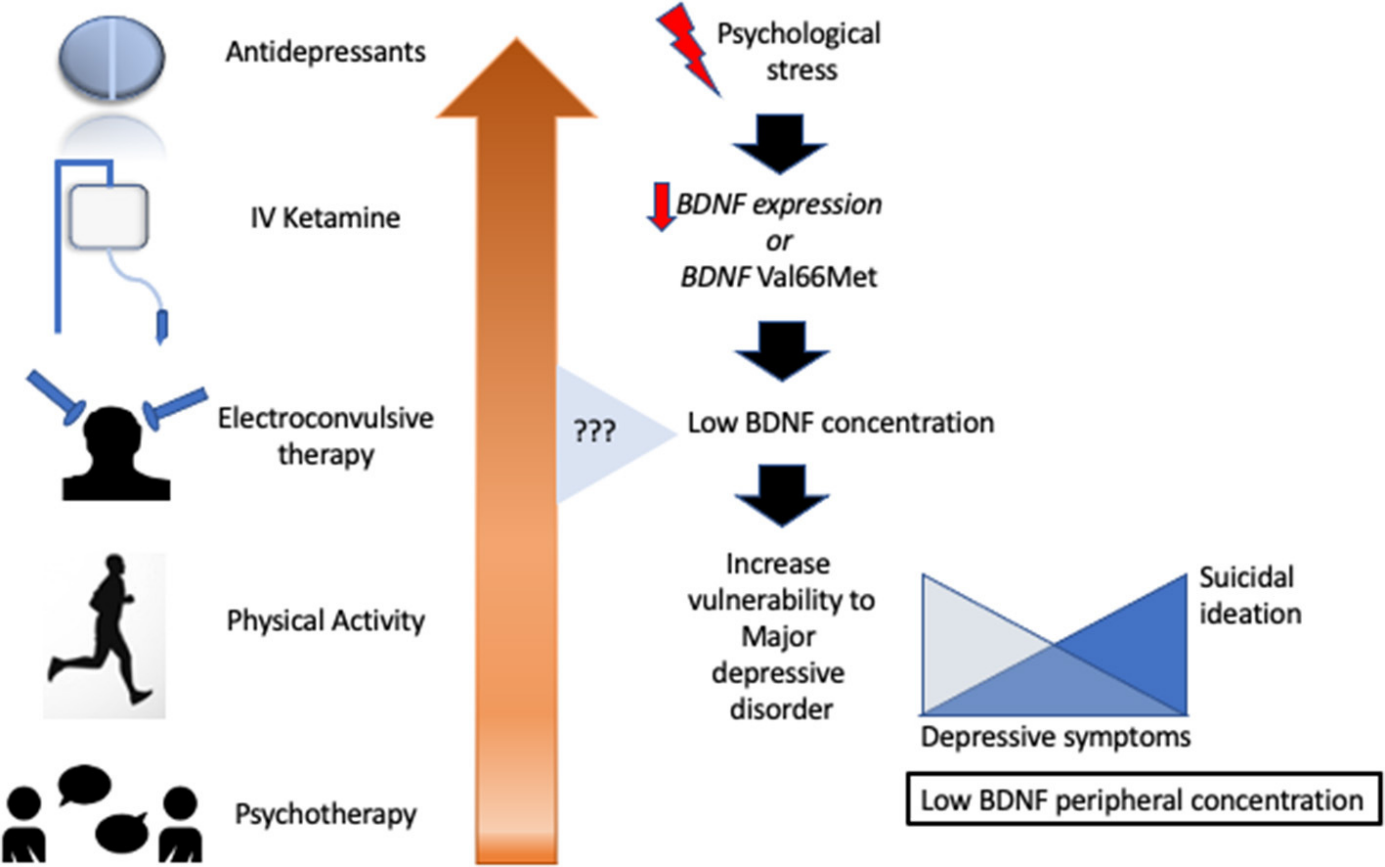
Several antidepressant treatments aimed to increase brain-derived neurotrophic factor with heterogeneous results.

In summary, experimental and clinical evidence points to both blood BDNF levels (serum or plasma) and the *BDNF* Val66Met polymorphism as being linked to MDD pathophysiology and treatment response. Taken together, several studies have indicated the blunt expression of BDNF in carriers of the Val66Met gene, lower BDNF levels in depressed individuals, and increased levels of BDNF after antidepressant therapy, especially involving SSRIs, ECT, and rapid-acting antidepressants such as ketamine (Figure 2). However, the high levels of heterogeneity, publication biases, and lower sample sizes of the studies in this area prevent an extrapolation of this compelling evidence to clinical practice, resulting in a low level of translational evidence. Moreover, technical issues still negatively influence our trust in peripheral measures of BDNF and its real correlation to brain levels. Several concerns remain to be answered regarding the association between BDNF levels and its (1) relationship with MDD severity, remission, and relapse; (2) direct effects vs. augmented expression of BDNF after antidepressants; (3) long-term antidepressant therapy maintenance; (4) response to other treatment options (e.g., physical activity and psychotherapy); (5) variation according to age, sex, and ethnicity, among other important questions; and (6) ideal measurement of BDNF. To address this latter issue, we recommend the serum or serum: whole blood level of BDNF as a more consistent measure of its peripheral concentration.

Finally, especially considering antidepressant treatment, BDNF may only be a part and a multilevel action of known antidepressants to date. A new hypothesis for antidepressant action, namely, the undirected susceptibility to change, presumes that antidepressant treatment does not directly improve mood, but rather creates a favorable condition through increased brain plasticity to the action of the environment, which is fundamental to ensure recovery and is amplified by antidepressant action (Branchi, 2011; Castrén, 2013). Preclinical and clinical evidence, especially that focused on SSRIs, has shown that the higher levels of serotonin and better interactions of environmental effects, stress-related genes, or stressful environments can blunt this favorable action; healthy environments (higher social networks, employment) increase antidepressant responses; and psychotherapy that deals with the environment enhances psychopharmacological treatment (Branchi et al., 2013; Alboni et al., 2017).

In conclusion, future research must first better address laboratory techniques for peripheral BDNF peripheral detection with a high correlation with brain concentrations; second, it must minimize important heterogeneity factors such as the inclusion of different MDD phenotypes, different quality of patient's environment, the presence of child trauma, and genetic Val66Met polymorphisms; and third, it must explore potential direct BDNF effects depicted from rapid-acting antidepressants such as ketamine.

## Author Contributions

All authors contributed equally to the writing of this review.

## Conflict of Interest

The authors declare that the research was conducted in the absence of any commercial or financial relationships that could be construed as a potential conflict of interest.
